# Overexpression of *growth hormone* improved hepatic glucose catabolism and relieved liver lipid deposition in common carp (*Cyprinus carpio* L.) fed a high-starch diet

**DOI:** 10.3389/fendo.2022.1038479

**Published:** 2022-12-06

**Authors:** Yunya Wu, Rui Li, Xingxing Wu, Wei Guo, Wenrong Zhong, Yongming Li, Yanlong Song, Binbin Tao, Ji Chen, Dong Han, Shouqi Xie, Yaping Wang, Zuoyan Zhu, Wei Hu

**Affiliations:** ^1^ State Key Laboratory of Freshwater Ecology and Biotechnology, Institute of Hydrobiology, Innovation Academy for Seed Design, Chinese Academy of Sciences, Hubei Hongshan Laboratory, Wuhan, China; ^2^ College of Advanced Agricultural Sciences, University of Chinese Academy of Sciences, Beijing, China; ^3^ School of Basic Medical Science, Wuhan University, Wuhan, China; ^4^ Qingdao National Laboratory for Marine Science and Technology, Qingdao, China

**Keywords:** *growth hormone* overexpression, insulin sensitivity, glucose utilization, liver lipid deposition, common carp (*Cyprinus carpio* L.)

## Abstract

Growth hormone (GH) is important for regulating insulin secretion and carbohydrate metabolism, and its role in mammalian models of diabetes is relatively worked out. Although some fish species were used as models for diabetes research, the effects of GH on insulin and glucose catabolism and anabolism in these models remain to be clarified. In this study, we investigated the effect of GH on insulin and glucose catabolism and anabolism in an omnivorous fish using *GH* transgenic (T) common carp that consistently overexpressed *GH* and wild-type (WT) common carp. We compared the intestinal morphology, and digestive and absorptive capacity of fish fed commercial feed. We also analyzed the growth performance, insulin level, glucose catabolism and anabolism, lipid deposition, and lipid catabolism and anabolism in T carp and WT carp fed diets containing either 30% or 40% starch. In the intestine of T carp, α-amylase activity was enhanced, the number of goblet cells and intestinal villi surface area was increased, and the expression level of glucose transport protein-related genes (*glut2* and *sglt1*) was upregulated when compared to these indicators in WT carp. When fed either a normal or high-starch diet, the growth performance of T carp was better than that of WT carp. Compared with WT carp, serum insulin was increased and glucose was decreased, hepatic expression level of *igf-1* and glycolysis-related genes was increased, and the activity level of a hepatic enzyme related to glycolysis was enhanced in T carp. When fed with a high-starch diet, the serum alanine aminotransferase activity, hepatic lipid content, and malondialdehyde content were significantly lower in T carp than in WT carp. These results indicated that overexpression of *GH* (1) enhanced carbohydrate digestion and absorption in the carp intestine, (2) did not induce insulin resistance and improved glucose catabolism and utilization in carp, and (3) relieved liver lipid deposition. Our data might provide new insights into potential ways to improve glucose utilization in fish and diabetes treatments.

## 1 Introduction

Carbohydrates are a primary biological component. Carbohydrate metabolism, which includes carbohydrate digestion, fructose and galactose metabolism, glucose catabolism, mainly *via* glycolysis and the citric acid cycle, glycogenesis, glycogenolysis, the pentose phosphate pathway, and gluconeogenesis (1), is regulated by endocrine factors, such as growth hormone (GH), insulin, and insulin-like growth factor (IGF-1) (2).

Excessive food intake and a lack of physical exercise can lead to an imbalance in energy metabolism and excessive accumulation of carbohydrate and other energy substances, which may lead to obesity, diabetes, and other diseases (3). Although diabetes research models have been established in mammals such as mice (4), fish are an ideal model for diabetes research, as important signaling and metabolic pathways are conserved in fish, and various transgenic and mutant lines are available for large-scale genetic and drug screening (5). For example, the zebrafish (*Danio rerio*) model of diet-induced obesity has phenotypes similar to those of diabetes mellitus type 2, such as fasting hyperglycemia, hyperinsulinemia, and impaired glucose tolerance (6). Nile tilapia (*Oreochromis niloticus*) has been used as a model for diabetes mellitus type 1 research because of its resistance to the diabetogenic effect of streptozotocin (5). Cavefish (*Astyanax mexicanus*) are naturally insulin resistant and hyperglycemic, without any obvious adverse effects (7).

In addition to exploring the etiology of insulin resistance and diabetes-like symptoms using fish models, the effect of GH, which is an important factor regulating carbohydrate metabolism, on glucose level has also been studied in fish. For example, intraperitoneal injection of GH caused hyperglycemia in Mozambique tilapia (*Oreochromis mossambicus*) and coho salmon (*Oncorhynchus kisutch*) (8, 9), and treatment with GH decreased the glycolytic potential in the liver of rainbow trout (*Oncorhynchus mykiss*) (10). However, the detailed *in vivo* effects of GH on insulin and glucose catabolism and anabolism in fish need to be clarified.

In our laboratory, we developed a transgenic common carp (*Cyprinus carpio* L.) that consistently and stably overexpresses exogenous *GH* (11, 12). In the present study, we used this model to study the effect of GH on insulin and glucose catabolism and anabolism in omnivorous fish. We compared the intestinal morphology, feed digestion, and absorptive capacity of the *GH* transgenic (T) carp to wild-type (WT) carp fed a normal starch diet and found that overexpression of *GH* enhanced carbohydrate digestion and absorption in the carp intestine. We then compared insulin level and glucose catabolism and anabolism in T and WT carp fed either a normal diet or a high-starch diet and found that overexpression of *GH* did not lead to insulin resistance, but rather improved glucose catabolism and utilization. Comparison of the growth performance, lipid deposition, and lipid catabolism and anabolism of the T and WT carp fed either a normal diet or a high-starch diet showed that overexpression of *GH* improved growth performance and relieved liver lipid deposition, even under high-starch nutrition.

## 2 Materials and methods

### 2.1 Animals and experiment designs

The fry of T carp that consistently and stably overexpress exogenous *GH*, with higher serum GH level (13) and higher expression level of *gh* in various tissues, were hatched with WT carp fry on the same day, and were fed commercial aquafeed (Tongwei, China, 33% protein, 6% fat, 22% carbohydrate) twice a day at the Liangzi Lake Breeding Base of the Institute of Hydrobiology, Chinese Academy of Sciences (Wuhan, China).

Two experiments (1, Comparison of intestinal development between T carp and WT carp. 2, comparison of effect of different dietary starch level on the growth and glucose metabolism between two strains) were performed. The fish used in Experiment 1 were fed commercial aquafeed till sampling. The fish used in Experiment 2 were transferred from the Liangzi Lake Breeding Base into an indoor recirculating aquaculture system at the age of 2 months, then fed normal-carbohydrate (30% carbohydrate, 33% protein, 6% fat) or high-carbohydrate (40% carbohydrate, 33% protein, 6% fat) diets during the growth experiment.

### 2.2 Experiment 1

#### 2.2.1 Analysis of intestinal development

WT and T carp were sampled at 45, 75, 105, 135, 165, and 195 days post fertilization (dpf) (8 individuals at every time point, respectively). After the fish fasted for 48 hours, the intestine was empty, and the fish bodies and intestines were weighed to calculate the intestinal somatic index (ISI) using the following formula:


ISI(%)=(intestine weight/body weight)×100

#### 2.2.2 Sampling for intestinal histology observations, enzyme activity testing, and analysis of nutrient transport gene expression level

4-month-old (as old as the fish after Experiment 2) T (292.1 ± 14.17 g) and WT (252.94 ± 14.72 g) carp were selected, and samples of the foregut, midgut, and hindgut were collected for analyses of intestinal enzyme activity, expression level of genes involved in nutrient transport, and intestinal morphology and histology. The sampling point of the foregut was about 0.3-0.5 cm before the first fold, the sampling point of the midgut was at the middle of the intestine, and the sampling point of the hindgut was about 0.3-0.5 cm after the last fold. After the fish fasted for 6 hours (expression level of genes involved in nutrient transport reached the highest at this point compared with 3 hours, 12 hours, and 24 hours, data unpublished), the foregut, midgut and hindgut (about 0.3 cm) of T carp and WT carp (n=6) were taken separately, the intestinal wall was cut with scissors, and the samples were rinsed in phosphate buffered saline (PBS) until there were no intestinal contents, and then placed in EP tubes, quickly frozen in liquid nitrogen and stored at -80°C for later gene expression analysis. After the fish fasted for 6 hours, the foregut, midgut and hindgut (about 0.5 cm) of T carp and WT carp (n=6) were taken separately (include intestinal wall and intestinal contents) and placed in EP tubes, quickly frozen in liquid nitrogen and stored at -80°C for later enzyme activity analysis. After the fish fasted for 48 hours, the intestine was empty. The foregut, midgut and hindgut (about 0.5 cm) of T carp and WT carp (n=6) were taken separately and fixed in Bouin’s solution overnight, then stored in 70% alcohol at 4°C for later morphology and histology analysis.

#### 2.2.3 Analysis of intestinal enzyme activity

The tissue samples were added with PBS in the ratio of weight (g): volume (mL) = 1:9, homogenized mechanically in an ice-water bath. Then the mixture was centrifuged at 3,000 rpm for 10 min, and the supernatant was collected for microplate reader analysis. The activities of intestinal enzymes in the carp after fasting for 6 hours were measured using commercial assay kits (Nanjing Jiancheng Biotech Co., Ltd., China) according to the manufacturer’s protocols. The activities of α-amylase (α-AMS), lipase (LPS), chymotrypsin, trypsin, and protein content were analyzed by using colorimetric methods and measuring the absorbance at 660, 580, 660, 253, and 562 nm, respectively, using a microplate reader (FlexStation 3; Molecular Devices, USA). The data shown were from three independent replicates.

#### 2.2.4 Analysis of intestinal transport gene expression level

Total RNA was extracted from the intestine of carp after fasting for 6 hours using TRIzol reagent (Invitrogen, USA) according to the manufacturer’s protocol. After treatment with DNase I (Promega, USA), 1 μg of total RNA was reverse transcribed using the ReverTra Ace M-MLV kit (TOYOBO, Japan). The cDNA sample was diluted 10-fold with RNase-free water and then used as a template for qPCR on a Bio-Rad CFX384 Real-Time PCR System (Bio-Rad, USA). Each reaction contained 5 µL of 2× SYBR Green Realtime PCR Master Mix (Toyobo, Japan), 0.5 µL (10 µM) of forward primer, 0.5 µL (10 µM) of reverse primer, 2 µL of H_2_O, and 2 µL of cDNA. The reaction conditions for qPCR were 95°C for 2 min, followed by 40 cycles of 95°C for 15 sec, 60°C for 20 sec, and 72°C for 30 sec. Relative mRNA expression level was calculated using the 2^-ΔΔCt^ method:


ΔCT=CT(target)−CT(β−actin)

The *β-actin* gene was used as the reference gene, and the calibrator sample was WT carp. Primer sequences and primer efficiencies were listed in **Supplementary Material 1**.

#### 2.2.5 Analysis of intestinal morphology and histology

The dehydrated intestine samples were permeabilized in xylene, and embedded in paraffin. Next, the embedded tissue block was cut into 5 μm slices. The slices were subjected to periodic acid-Schiff (PAS) staining, examined under a microscope equipped with a CCD (Aperio Versa 8 Scanner, Leica, Germany). Villi height, villi width, and length of enterocyte in the foregut, midgut, and hindgut in five randomly selected villi were analyzed using Aperio ImageScope software (Leica, Germany), with a scale corresponding to the photographic magnification. Intestinal goblet cell number was measured by counting cells in the foregut, midgut, and hindgut in five randomly selected villi.

### 2.3 Experiment 2

Normal carbohydrate (NC, 30% C) and high-carbohydrate (HC, 40% C) diets were prepared, and the formulations and chemical compositions were listed in **Supplementary Material 2**. White fish meal, soybean meal, and casein were the major protein sources; fish oil and soybean oil were the major sources of fat; corn starch was the carbohydrate source. All raw materials were sieved through 40-mesh, mixed, and then formed into 2 mm pellets using a laboratory extruder (Mechanical Facility Research Institute, China). The pellets were dried at 70°C and then stored at 4°C.

Similar-sized, 2-month-old WT (3.29 ± 0.20 g) carp and T (3.57 ± 0.44 g) carp were weighed and distributed into six tanks (diameter: 80 cm, water volume: 400 L) with 30 fish per tank in a recirculating aquaculture system. Each strain was randomly assigned to six tanks. All fish fasted for 24 hours before the growth experiment. Then, three tanks of T and WT carp each were fed the NC diet, while three tanks of T and WT carp each were fed the HC diet, forming four groups (three replicate tanks in each group), which were fed to satiation twice a day (at 9:00 and 17:00) for 8 weeks. The daily feed intake of the fish in each tank was recorded. During the experiment, the water temperature was maintained at 29.9°C–30.9°C, the dissolved oxygen was at >6.0 mg/L, and the ammonia nitrogen concentration was at<0.4 mg/L. The tanks were housed under a 12 h light: 12 h dark photoperiod, with light from 7:00 to 19:00.

After 8 weeks, all fish were weighed to calculate weight gain (WG), specific growth rate (SGR), and feed efficiency (FE) using the following formulas:


WG(%)=100×[final body weight(g)−initial body weight(g)]/initial body weight(g)

SGR(%/d)=100×{Ln[final body weight(g)]−Ln[initial body weight(g)]}/d

FE(%)=100×[final body weight(g)−initial body weight(g)]/dry feed fed(g)

#### 2.3.1 Sampling for analysis of biochemical parameters

After the growth experiment, all fish fasted overnight. Six fish from each group (two fish per tank) were randomly selected and anesthetized with 40 mg/L ethyl 3-aminobenzoate methane sulfonate (MS-222; Macklin, China) for blood and liver sampling. The blood was drawn from caudal vein using a 1 mL syringe, settled at 4°C for 4 hours, then centrifuged at 4°C by 1,500 rpm for 30 min. The serum was isolated and stored at −80°C for later biochemical analysis. Each liver sample was placed into four tubes, immediately frozen in liquid nitrogen, and then stored at −80°C for subsequent biochemical analysis, RNA isolation, and histological analysis.

#### 2.3.2 Measurement of glucose catabolism- and anabolism-related biochemical parameters

The biochemical parameters of six fish from each group (two fish per tank) were determined using commercial assay kits (Nanjing Jiancheng Biotech Co., Ltd., China) according to the manufacturer’s protocols. Serum glucose concentration was determined using the glucose oxidase method (absorbance at 340 nm). The concentrations of glycogen, pyruvate, and lactate in the liver were determined using the anthranone method (absorbance at 620 nm), colorimetric method (absorbance at 505 nm), and lactate dehydrogenase catalysis method (absorbance at 530 nm), respectively. The activities of hexokinase (HK), pyruvate kinase (PK), and phosphoenolpyruvate carboxylase kinase (PEPCK) in the liver were determined using a colorimetric method (absorbance at 340 nm). Absorbance was measured using a microplate reader (FlexStation 3; Molecular Devices, USA). The data shown was from three independent replicates.

Insulin level in the serum of three fish from each group (one fish per tank) was measured using a mass spectrometer (Triple TOF 6600+, Sciex, USA). The serum samples were pre-treated as follows: 200 µL of 8 M urea was added to the serum and sonicated in an ice bath for 5 min. After the serum was centrifuged at 4°C and 12,000 *g* for 15 min, the concentration of total protein in the supernatant was determined using the Bradford Protein Assay Kit (Sangon Biotech, China). Each sample (containing 200 µg of protein) was oxido-reduced by incubation with 25 mM dithiothreitol at 37°C for 45 min and then alkylated by incubation with 50 mM iodoacetamide in the dark for 15 min. Next, 100 mM Tris-HCl was added to dilute the urea in the sample to<2 M, and trypsin (Promega, USA) was added to digest the proteins (37°C, 20 hours). Trifluoroacetic acid was added to a final concentration of 0.1% to stop protein digestion. After centrifugation at 12,000 *g* for 15 min, the supernatant was subjected to peptide purification using a Sep-Pak C18 desalting column (55–105 µm, 125 Å pore size; Waters, USA). The desalted peptide solution was dried at 45°C in a SpeedVac (Thermo, USA) for subsequent mass spectrometer analysis. Data were analyzed using the target quantitative software Skyline Daily (v20.2.0.286) and calculated using MultiQuant 3.0.3 software (AB Sciex). For the quantification of insulin, the abundance was calculated by summation of the peak areas of their corresponding peptides.

#### 2.3.3 Measurement of liver health-related biochemical parameters

Serum activities of alanine aminotransferase (ALT) and aspartate aminotransferase (AST) were determined using the Reitman-Frankel method (absorbance at 510 nm). Malondialdehyde (MDA) content in the liver was determined using the TBA method (absorbance at 532 nm). The data shown was from three independent replicates.

#### 2.3.4 Hepatic fat content determination

Six liver samples from each group (two fish per tank) were fixed in 4% PFA at 4°C overnight. After dehydration with 30% sucrose, the samples were embedded in the O.C.T. compound and cryosectioned. The sections were then stained with Oil Red O to visualize fat deposits. Images were observed and captured using a microscope equipped with a CCD (Aperio Versa 8 Scanner; Leica, Germany), and the relative area of the lipid droplets was analyzed using Image-Pro Plus 6.0. Averages from 4–6 pictures were reported.

Total lipid in liver samples from six individuals in each group (two fish per tank) was extracted and determined using the chloroform/methanol (2:1, v:v) method (14). The data shown was from three independent replicates.

#### 2.3.5 Analysis of hepatic glucose and lipid catabolism- and anabolism-related genes expression level

Total RNA was extracted from liver samples from six individuals in each group (two fish per tank) as described as above. The qPCR reaction conditions were the same as above, the calibrator sample was WT carp fed NC diet, and the primer sequences were listed in **Supplementary Material 1**. The data shown was from three independent replicates.

#### 2.3.6 Glucose tolerance test

After the growth experiments, 30 fish from each group (ten fish per tank) were selected, fasted overnight, anesthetized, and then intraperitoneally injected with D-glucose (Sigma, Germany) at 500 mg/kg body weight (20% glucose dissolved in 0.85% NaCl). Blood was collected from five fish in each group at 0, 0.5, 1, 3, 6, and 12 hours after glucose injection. Serum glucose concentration was determined using commercial assay kits, as described above.

### 2.4 Statistical analyses

All data was presented as the mean ± standard error of the mean (SEM) and was analyzed using SPSS statistics software (version 22.0; IBM, USA). Data related to intestinal histology, digestive enzyme activities, and expression level of genes involved in nutrient transport was analyzed using independent-samples *t*-test. GTT data was analyzed using one-way analysis of variance (ANOVA) followed by Tukey’s HSD multiple range test. GTT data between different groups at the same sampling time was also analyzed using one-way ANOVA. All other data was analyzed using independent-sample *t*-test to estimate the significance of differences between T and WT carp fed the same diet or between NC and HC treatments for the same strain. Data was further analyzed using two-way ANOVA to determine the interaction between *GH* level and dietary carbohydrate level. Statistical significance was set at *P*< 0.05.

## 3 Results

### 3.1 Effect of *GH* overexpression on intestinal histology, digestive enzyme activities, and expression level of the genes involved in nutrient transport

**Figure 1:**
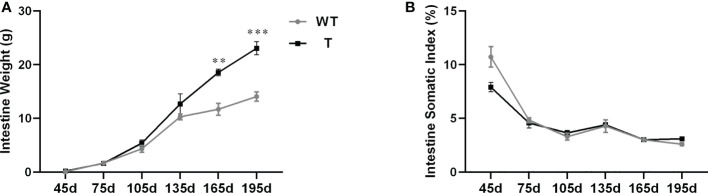
Intestinal development of transgenic (T) common carp (*Cyprinus carpio* L.) that overexpress *GH* and wild-type (WT) common carp from age 45 to 195 days. **(A)** Intestine weight (n = 8). **(B)** Intestine somatic index (n = 8). **P < 0.01, ***P < 0.001.

The intestinal weight of T and WT carp from 45 to 135 dpf did not differ. However, after 165 dpf, the intestinal weight of T carp was significantly higher than that of WT carp (*P*< 0.05; **Figure 1A**). There was no significant difference in ISI between two strains (**Figure 1B**).

**Figure 2:**
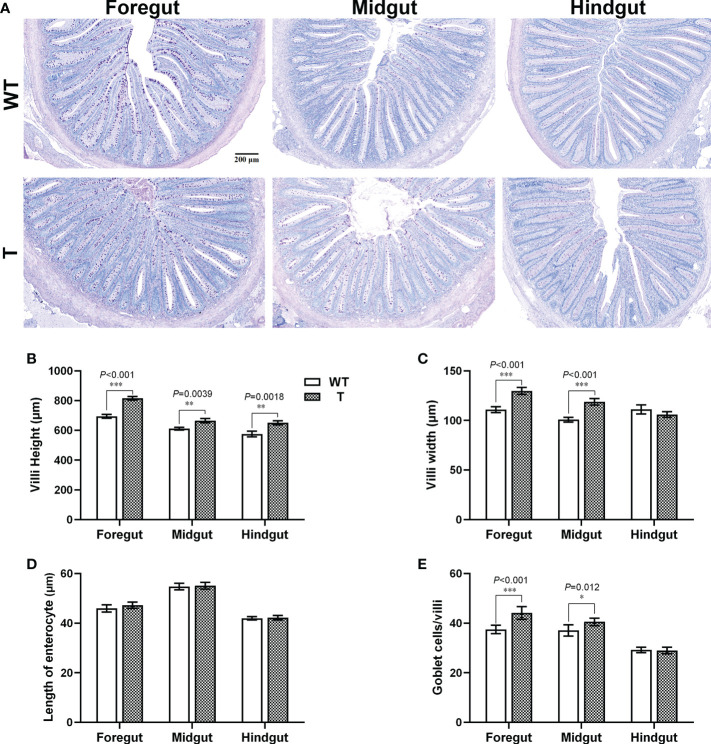
Intestinal histology of 4-month-old T and WT common carp. **(A)** Intestinal sections stained with PAS. Scale bars, 200 μm (7×). The arrows indicate goblet cells. **(B–E)** Villus height, villus width, length of enterocyte, and the number of goblet cells per villus in the intestine (n = 6). WT, wild-type common carp. T, *GH* transgenic common carp. *P < 0.05, **P < 0.01, ***P < 0.001.

**Figure 3:**
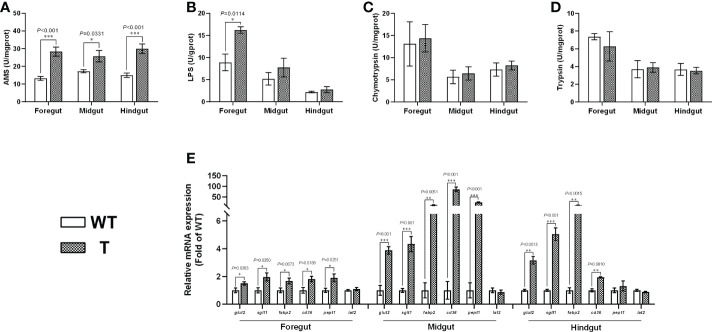
Intestinal digestive enzyme activity levels and the gene expression levels of nutrient transport proteins in 4-month-old T and WT common carp. **(A-D)** Activity levels of α-amylase (α-AMS), lipase (LPS), chymotrypsin, and trypsin. **(E)** The gene expression levels of nutrient transport proteins (n = 6). WT, wild-type common carp. T, *GH* transgenic common carp. *P < 0.05, **P < 0.01, ***P < 0.001.

Intestine is an important organ for nutrient digestion and absorption. Based on the PAS staining results, the villi heights in the foregut, midgut, and hindgut of T carp were significantly higher than those of WT carp (*P*< 0.05), the villi width in the foregut and midgut of T carp was significantly wider than that of WT carp (*P*< 0.05), and no significant difference was observed in length of enterocyte in the foregut, midgut, and hindgut between two strains (**Figures 2A–D**), indicating that the surface area of the intestinal villi in T carp was larger than that in WT carp. Goblet cells in intestinal villi secrete mucus to facilitate digestion. Based on the PAS staining results, the foregut and midgut of T carp contained significantly more goblet cells than WT carp (*P*< 0.05; **Figures 2A, E**). The activity of α-AMS in the foregut, midgut, and hindgut of T carp was significantly higher than WT carp (*P*< 0.05), and LPS activity in the foregut was significantly higher in T carp than in WT carp (*P*< 0.05). In contrast, the activities of chymotrypsin and trypsin were not significantly different (**Figures 3A–D**). The expression level of the glucose transport protein-related genes (*glut2* and *sglt1*) and the fatty-acid transport protein-related genes (*fabp2* and *cd36*) in the intestine was significantly higher in T carp than in WT carp, and the expression level of the peptide transport protein-related gene *pept1* in the foregut and midgut was significantly higher in T carp than in WT carp (*P*< 0.05; **Figure 3E**).

### 3.2 Effect of *GH* overexpression on the growth performance of carp fed diets with different carbohydrate level

**Figure 4:**
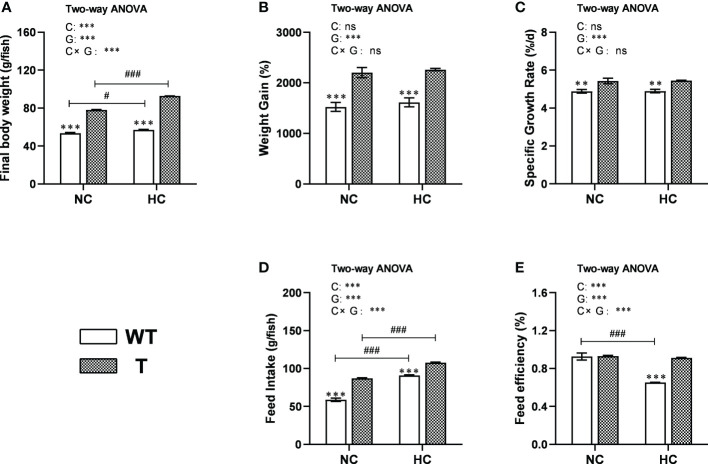
Growth performance of T and WT common carp fed a normal starch or high-starch diet. **(A)** Final body weight. **(B)** Weight gain. **(C)** Specific growth rate. **(D)** Feed intake. **(E)** Feed efficiency (n = 3). WT, wild-type common carp. T, *GH* transgenic common carp. NC, diet containing 30% starch. HC, diet containing 40% starch. ** and *** indicate a significant difference (*P* < 0.01, and *P* < 0.001, respectively) in different strains at same dietary starch content. # and ### indicate a significant difference (*P* < 0.05 and *P* < 0.001, respectively) in the same strain with different dietary starch contents. In two-way ANOVA, C, carbohydrate; G, GH. ***P < 0.001, ns indicates no significance.

When fed either NC or HC diet, the final body weight and feed intake of T carp were significantly higher than WT carp (*P*< 0.05). When fed a higher level of dietary starch, the final body weight and feed intake of both stains increased significantly (*P*< 0.05; **Figures 4A, D**). When fed either NC or HC diet, the WG and SRG of T carp were significantly higher than WT carp (*P*< 0.05), and these values did not change with dietary starch level (**Figures 4B, C**). When fed NC diet, there was no difference in FE between two strains. However, when the dietary starch level was higher, the FE of WT carp decreased significantly (*P*< 0.05), while the FE of T carp did not change significantly. Therefore, when fed an HC diet, the FE of T carp was significantly higher than WT carp (*P*< 0.05; **Figure 4E**).

### 3.3 Effect of *GH* overexpression on glucose catabolism, anabolism, and utilization and insulin sensitivity in carp fed diets with different carbohydrate level

**Figure 5:**
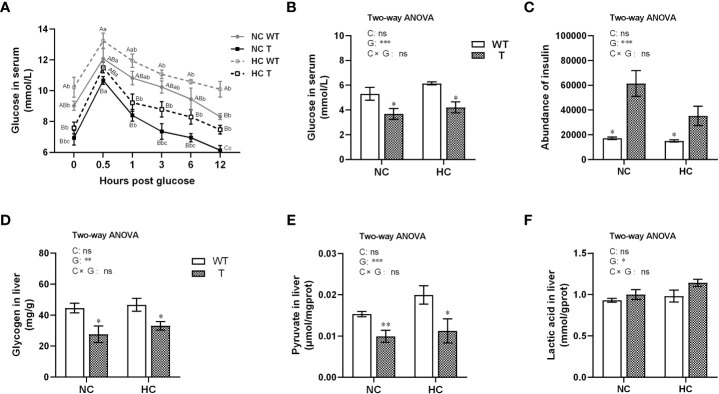
Glucose utilization in T and WT common carp fed a diet with normal or high-carbohydrate content. **(A)** Serum glucose levels in intraperitoneal glucose tolerance test (GTT) (n = 5). **(B, C)** Serum glucose (n = 6) and insulin (n = 3) levels. **(D-F)** Glycogen, pyruvate, and lactic acid contents in the liver (n = 6). In the GTT, different lowercase letters indicate significant differences (*P*< 0.05) at different time points within each group, whereas different uppercase letters indicate significant differences (*P*< 0.05) among different groups at the same sampling time point. * and ** indicate a significant difference (*P* < 0.05 and *P* < 0.01, respectively) in different strains at same dietary starch content. In two-way ANOVA, C, carbohydrate; G, GH. *P < 0.05, **P < 0.01, ***P < 0.001, ns, indicates no significance.

In the GTT experiment, plasma glucose concentration in T and WT carp increased significantly (*P*< 0.05), peaked at 0.5 hour, then decreased gradually over the next 12 hours. During the 0.5–1 h period, plasma glucose concentration dropped faster in T carp than in WT carp (**Figure 5A**). In the resting state (fasted overnight), when fed either NC or HC diet, T carp had lower serum glucose concentration, lower hepatic glycogen and pyruvate content, and higher serum insulin level than WT carp (*P<* 0.05). There was no significant difference in hepatic lactate acid content between two strains. When dietary starch content increased, there were no significant differences in serum glucose concentration, insulin level, and content of glycogen, pyruvate, and lactate acid in the liver between two strains (**Figures 5B–F**).

**Figure 6:**
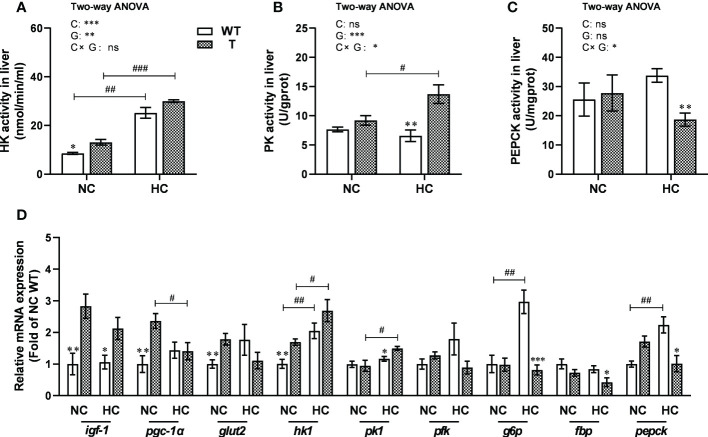
Hepatic enzymatic activity and expression levels of genes involved in carbohydrate metabolism in T and WT common carp fed a normal or high-carbohydrate diet. **(A-C)** Activity levels of hexokinase (HK), pyruvate kinase (PK), and phosphoenolpyruvate carboxylase kinase (PEPCK) in the liver. **(D)** The expression levels of genes involved in carbohydrate metabolism in the liver (n = 6). *, ** and *** indicate a significant difference (*P* < 0.05, *P* < 0.01, and *P* < 0.001, respectively) in different strains at same dietary starch content. #, ## and ### indicate a significant difference (*P* < 0.05, *P* < 0.01, and *P* < 0.001, respectively) in the same strain with different dietary starch contents. In two-way ANOVA, C, carbohydrate; G, GH. *P < 0.05, **P < 0.01, ***P < 0.001; ns, indicates no significance.

When fed NC diet, the activity of hepatic HK, a key enzyme in glycolysis, was significantly higher in T carp than WT carp (*P<* 0.05), whereas the activities of hepatic PK and PEPCK, the key enzymes in glycolysis and gluconeogenesis, respectively, did not change significantly. When fed HC diet, the activity of hepatic PK was significantly higher in T carp than WT carp (*P<* 0.05), whereas the activity of hepatic PEPCK was significantly lower in T carp than WT carp (*P<* 0.05). The activity of hepatic HK was not significantly different. When the dietary starch content increased, the activities of hepatic HK and PK increased significantly in T carp (*P<*0.05), whereas the activity of hepatic PEPCK did not change significantly. The activity of hepatic HK increased significantly in WT carp (*P<* 0.05), whereas the activities of hepatic PK and PEPCK did not change significantly (**Figures 6A–C**).

When fed NC diet, the expression level of *igf-1*, *pgc-1α*, *glut2*, and *hk1* was significantly higher in the liver of T carp than WT carp (*P<* 0.05). When fed HC diet, the expression level of *igf-1* and *pk1* was significantly higher (*P<* 0.05) and the expression level of *g6p*, *fbp*, and *pepck* was significantly lower (*P<* 0.05) in the liver of T carp than WT carp. When the dietary starch content increased, the expression level of *hk1* and *pk1* increased significantly (*P<* 0.05), and the expression level of *pgc-1α* decreased significantly (*P<* 0.05) in the liver of T carp. The expression level of *hk1*, *g6p*, and *pepck* increased significantly (*P<* 0.05) in the liver of WT carp (**Figure 6D** and **Supplementary Material 3**).

### 3.4 Effect of *GH* overexpression on lipid accumulation, liver health, and lipid catabolism and anabolism in the liver of carp fed diets with different carbohydrate level

**Figure 7:**
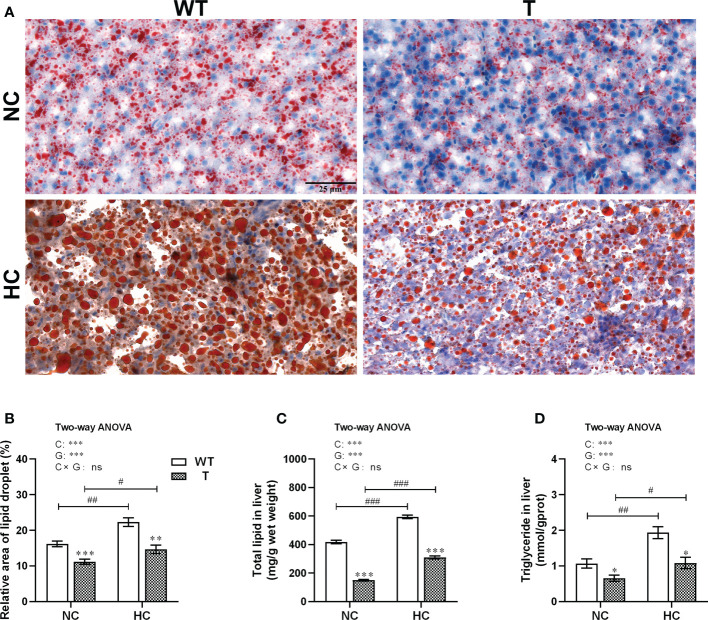
Lipid accumulation in the liver of T and WT common carp fed a normal or high-carbohydrate diet. **(A)** Liver sections stained with Oil Red O. Scale bars, 25 μm (40×). **(B)** Relative lipid droplet areas in the liver. **(C, D)** Total lipid and triglyceride contents in the liver (n = 6). *, ** and *** indicate a significant difference (*P* < 0.05, *P* < 0.01, and *P* < 0.001, respectively) in different strains at same dietary starch content. #, ## and ### indicate a significant difference (*P* < 0.05, *P* < 0.01, and *P* < 0.001, respectively) in the same strain with different dietary starch contents. In two-way ANOVA, C, carbohydrate; G, GH. ***P < 0.001; ns, indicates no significance.

Liver oil red O staining showed that the hepatic lipid droplet contents of both T and WT carp increased significantly (*P<* 0.05) when the dietary starch content increased. When fed either NC or HC diet, the relative area containing lipid droplets in the liver was significantly lower in T carp than WT carp (*P<* 0.05; **Figures 7A, B**). Similar conclusion could be drawn from the results obtained either using the chloroform/methanol extraction method to determine total lipid content in the liver (**Figure 7C**), or using the commercial kit for triglyceride content in the liver (**Figure 7D**).

**Figure 8:**
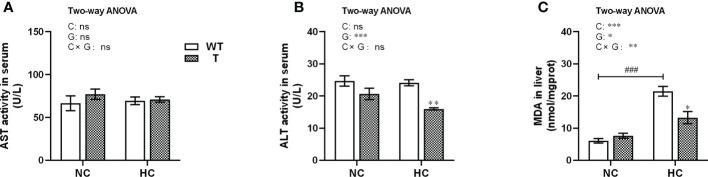
Indicators of liver damage of T and WT common carp fed a normal or high-carbohydrate diet. **(A, B)** Serum activity levels of aspartate aminotransferase (AST) and alanine aminotransferase (ALT). **(C)** Malondialdehyde (MDA) content in the liver (n = 6). * and ** indicate a significant difference (*P* < 0.05 and *P* < 0.01, respectively) in different strains at same dietary starch content. ### indicate a significant difference (*P* < 0.001) in the same strain with different dietary starch contents. In two-way ANOVA, C, carbohydrate; G, GH. *P < 0.05, **P < 0.01, ***P < 0.001; ns, indicates no significance.

When T and WT carp were fed NC diet, the AST and ALT activities in serum and the MDA content in liver were not significantly different. However, the MDA content in the liver of WT carp increased significantly (*P<* 0.05) with dietary starch content. When fed HC diet, the MDA content in liver and ALT activity level in serum were significantly lower in T carp than WT carp (*P<* 0.05; **Figures 8A–C**).

**Figure 9:**
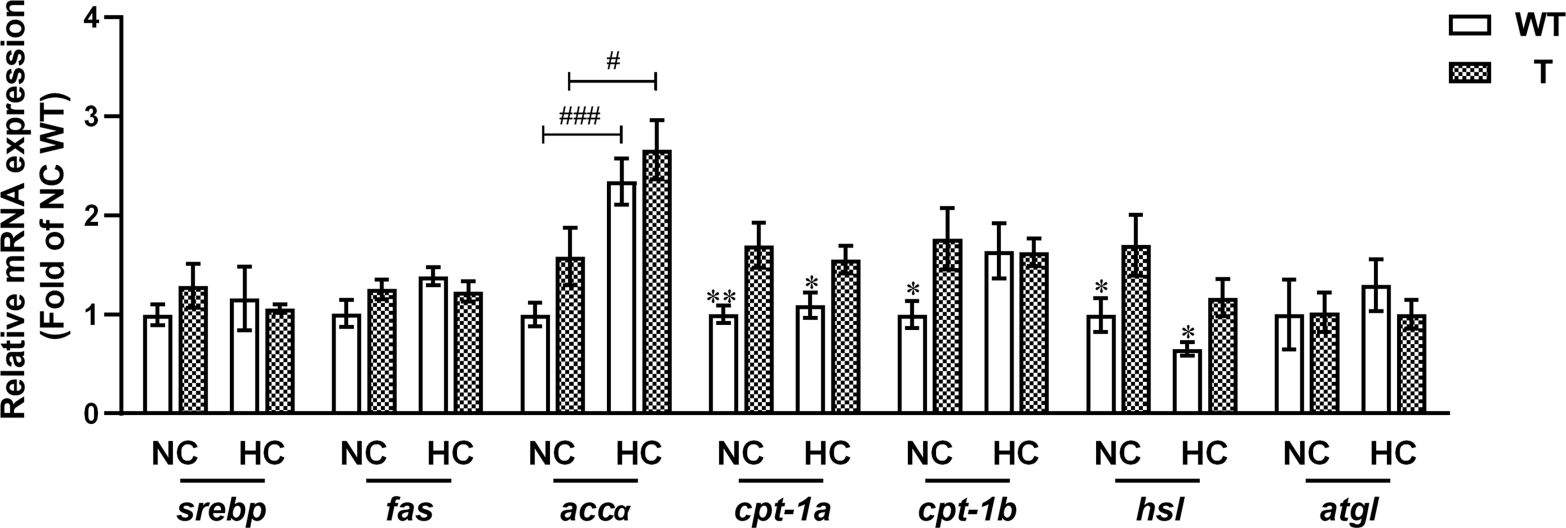
Expression levels of genes involved in lipid metabolism in the liver of T and WT common carp (n = 6). * and ** indicate a significant difference (*P* < 0.05 and *P* < 0.01, respectively) in different strains at same dietary starch content. # and ### indicate a significant difference (*P* < 0.05 and *P* < 0.001, respectively) in the same strain with different dietary starch contents.

The qPCR analysis showed that the expression level of *cpt1a*, *cpt1b*, and *hsl* in liver was significantly higher in T carp than WT carp when fed NC diet (*P<* 0.05). The expression level of *cpt1a* and *hsl* in liver was significantly higher in T carp than WT carp when fed HC diet (*P<* 0.05). When the dietary starch content increased, the expression level of *accα* increased significantly in the liver of both T and WT carp (*P<* 0.05; **Figure 9** and **Supplementary Material 4**).

## 4 Discussion

In this study, we found that in a transgenic common carp family that overexpressed *growth hormone*, the intestinal digestion, absorption of carbohydrates, and hepatic glucose catabolism and utilization were enhanced. When fed a high-starch diet, T carp had higher WG, SGR, and FE, lower lipid and MDA content in liver, and lower serum ALT activity than WT carp.

### 4.1 Overexpression of *GH* in common carp enhanced carbohydrate digestion and absorption

Vertebrate gastrointestinal tract is a flexible system, and its size, structure, and functional properties can change to meet functional demands (15). In most animals, intestinal absorption is influenced by both villus surface area at tissue level and microvillus surface area at cellular level (16). In the present study, there was no significant difference in ISI between T and WT carp. However, the height and width of the villi increased in T carp, indicating that the villus surface area increased in T carp compared to WT carp. A similar phenomenon was observed in *GH* transgenic coho salmon (17). These results suggest that overexpression of *GH* alters the intestinal structure of fish to improve nutrient absorption. In addition, we found that overexpression of *GH* upregulated the expression level of the glucose transport protein-related genes (*glut2* and *sglt1*) and the fatty-acid transport protein-related genes (*fabp2* and *cd36*) in intestine and the expression level of the peptide transport protein-related gene *pept1* in foregut and midgut. This was also observed in GH-treated tilapia and *GH*-transgenic zebrafish (18, 19). The increased absorptive area and enhanced transport capacity could result in greater intestinal absorption of small molecular nutrients in T carp when compared to in WT carp.

Common carp is a stomach-less fish (20), and nutrient digestibility depends mainly on the activities of intestinal digestive enzymes such as α-AMS, LPS, and protease (21). In our study, α-AMS activity in the intestine was significantly higher in T carp than in WT carp. In addition, the numbers of goblet cells in foregut and midgut were significantly larger in T carp than in WT carp. Goblet cells in the intestine of fish can secrete mucus, which helps to protect the intestine and aids in nutrient digestion (22). Increased level of α-AMS activity and mucus could lead to improved carbohydrate digestion in T carp compared to WT carp.

### 4.2 Overexpression of *GH* in common carp relieved liver lipid deposition, likely caused by excessive hepatic lipid accumulation, by enhancing glucose catabolism and utilization

Previous studies showed that carnivorous fish, such as European Sea Bass (*Dicentrarchus labrax* L.) and Gilthead Sea Bream (*Sparus aurata* L.), were glucose intolerant, and when fed a high-carbohydrate diet, their serum glucose concentration and hepatic glycogen content increased (23). Common carp is an omnivorous species. In the present study, the concentration of serum glucose and insulin, and the contents of glycogen, pyruvate, and lactate in liver did not change with starch content in the diet. Similarly, previous studies on Gibel carp (*Carassius gibelio*) and grass carp (*Ctenopharyngodon idellus*) showed that serum glucose concentration did not change with dietary carbohydrate content (24, 25). These findings suggested that omnivorous fish like common carp might have higher glucose tolerance than carnivorous fish.

In mammals, overexpression of *GH* leads to insulin resistance along with high serum GH level (26, 27). It has been suggested that chronic excess GH expression impairs insulin sensitivity by increasing glucose production *via* induction of hepatic gluconeogenesis. On the other hand, GH increases free fatty acid production through lipolysis, leading to glucose-fatty acid substrate competition and decreased glucose utilization (28). In the present study, T carp had higher serum insulin level than WT carp, which is similar to mammals. However, T carp had lower serum glucose concentration than WT carp, without impairing insulin sensitivity, which might be explained as follows. First, the *igf-1* expression level in the liver was significantly higher in T carp than in WT carp. IGF-1, which is regulated by GH, increases glucose uptake and reduces serum glucose and hepatic glucose production (29). Second, we found that overexpression of *GH* enhanced glucose catabolism and utilization in the liver, as T carp had significantly faster glucose clearance rates and lower hepatic glycogen and pyruvate contents than WT carp. The activity of the glycolysis-related enzyme HK and its gene expression level in the liver were significantly higher in T carp than in WT carp when fed a normal starch diet. In addition, T carp had higher glycolysis-related enzyme (PK) activity and gene expression level and lower activity of the gluconeogenesis-related enzyme PEPCK and expression level of the gluconeogenesis-related genes *g6p*, *fbp*, and *pepck* in the liver than WT carp when fed a high-starch diet. It was also observed that glycolysis-related enzyme activities and gene expression level increased in the liver of *GH* transgenic tilapia (*Oreochromis* sp.), Atlantic salmon (*Salmo salar*), and coho salmon (30–33). These results suggested that overexpression of *GH* might enhance glycolysis in the liver of fish. In addition, when the dietary starch content increased, the FE of T carp did not change significantly, whereas the FE of WT carp decreased significantly. Moreover, T carp had better growth performance than WT carp when fed a high-starch diet, indicating that overexpression of *GH* in carp could enhance glucose utilization.

**Figure 10:**
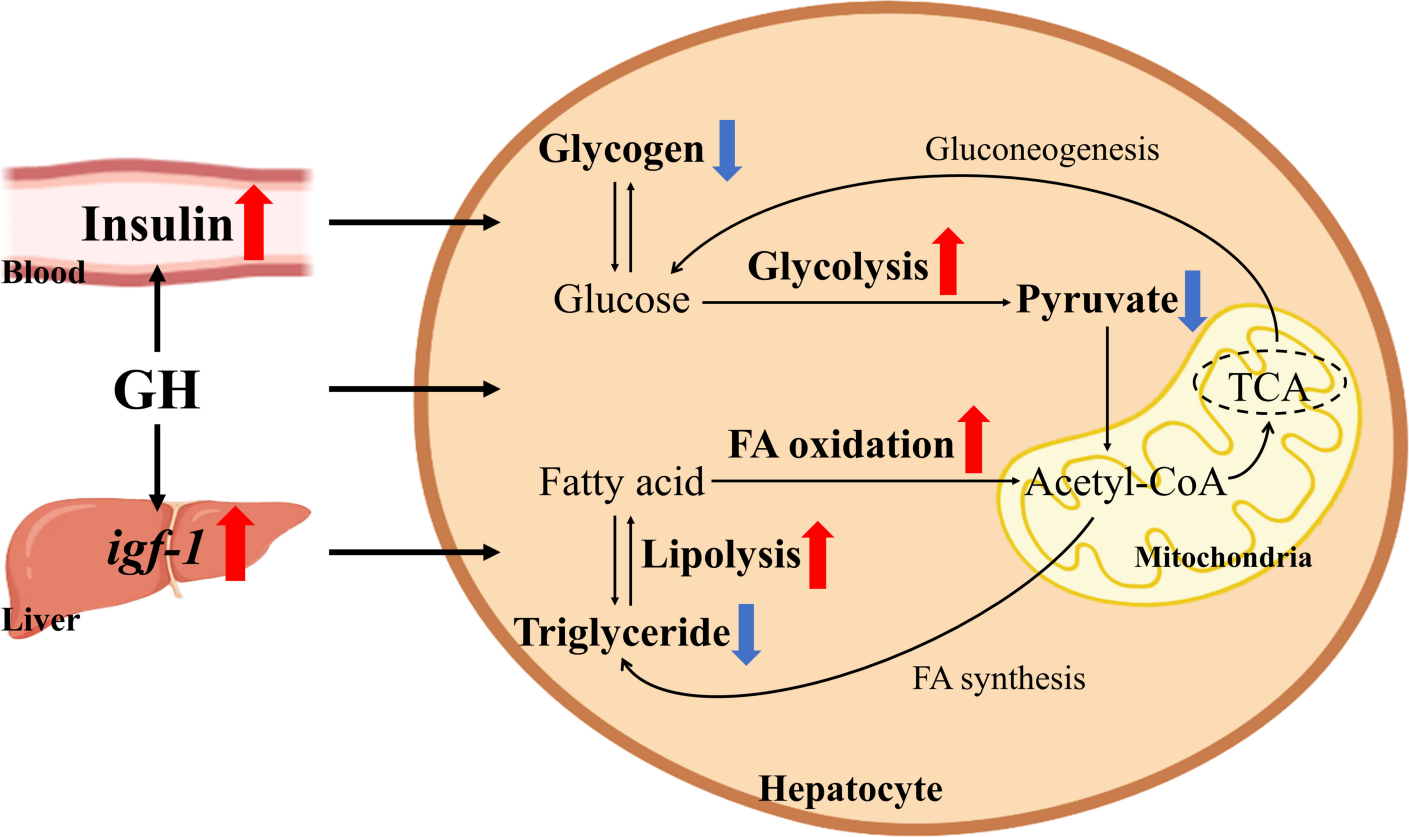
Schematic of the effects of *GH* overexpression on glucose and lipid catabolism in common carp. Red arrows indicate upregulated genes, proteins, or pathway. Blue arrows indicate decreased contents.

Both T and WT carp had higher expression level of the lipid synthesis-related gene *accα* in liver and higher lipid content when the dietary starch content increased. Excessive carbohydrate intake over time also caused hepatic lipid accumulation (34–36). However, the hepatic lipid content of T carp fed HC diet was significantly lower than that of WT carp fed HC diet and was equal to the hepatic lipid content of WT carp fed NC diet. In addition, hepatic MDA (a product of lipid peroxidation) content was significantly higher in the liver of WT carp fed HC diet than in the liver of WT carp fed NC diet. When fed the HC diet, T carp had lower hepatic MDA content and serum ALT activity level than WT carp. A previous study suggested that lipid accumulation in hepatocytes increased vulnerability to oxidative stress and inflammatory cytokines, which were triggered by lipid peroxidation in the liver (37). These findings suggest that overexpression of *GH* can relieve hepatic lipid accumulation, and perhaps even liver damage, induced by excessive starch intake. This effect can be explained as follows. First, as previously described, T carp had lower hepatic intermediate metabolite content from glucose (pyruvate) and stronger glucose utilization than WT carp when fed HC diet, which possibly reduced largely lipogenesis from glucose. Second, hepatic lipid catabolism and utilization were enhanced in T carp. In this study, hepatic expression level of the fatty acid β-oxidation-related gene *cpt1a* and the lipolysis-related gene *hsl* was significantly higher in T carp than in WT carp when fed HC diet. Our recent study also showed that lipid contents in the liver, serum, and whole body were significantly reduced in T carp (38). In that study, we found overexpression of *GH* enhanced lipid catabolism and utilization in the liver by upregulating lipolysis (as evidenced by the activities of the lipolytic enzymes hormone-sensitive lipase and adipose triglyceride lipase, and by the expression level of *hsl*) and fatty acid β-oxidation pathways (as shown by the number of mitochondria and the expression level of *cpt1a* and *cpt1b*), which decreased lipid content in the liver. Lipid catabolism can produce energy for growth. Therefore, T carp showed better growth performance when fed a high-fat diet. In the present study, we found that overexpression of *GH* did not lead to insulin resistance but reduced serum glucose concentration and increased hepatic expression level of *igf-1* and glycolysis pathways, which decreased glycogen content in the liver. Carbohydrate catabolism can produce energy for growth. Therefore, T carp had better growth performance than WT carp when fed a high-starch diet. GH affects both glucose and lipid metabolism. Glucose and lipid can be converted to each other. Based on our results, we proposed a mechanism in which overexpression of *GH* reduced hepatic lipid content by enhancing hepatic glucose and lipid catabolism, which was shown in **Figure 10**.

In conclusion, our study demonstrated that overexpression of *GH* in common carp alleviated the adverse effects induced by a high-starch diet through promoting glucose catabolism and utilization without inducing insulin resistance, as usually was not observed in mammals. This study might provide new insights into the improvement of glucose utilization in fish and potential diabetes treatment.

## Data availability statement

The original contributions presented in the study are included in the article/**Supplementary Materials**. Further inquiries can be directed to the corresponding authors.

## Ethics statement

The animal study was reviewed and approved by Laboratory animal welfare and ethics committee, Institute of hydrobiology, Chinese Academy of Sciences.

## Author contributions

ZZ and WH contributed to the conception, supervision, and funding acquisition of the study. YYW, JC and WH wrote the manuscript. YYW, RL, WG, WZ, YL, DH, SX, YPW and WH performed the experiments. YYW, XW, YS, BT, JC and WH did data analysis. All authors read and approved this manuscript.
