# Supplementary material for: Overexpression of growth hormone improved hepatic glucose catabolism and relieved liver lipid deposition in common carp (Cyprinus carpio L.) fed a high-starch diet
Source: Front Endocrinol (Lausanne). 2022 Dec 6;13:1038479. doi: 10.3389/fendo.2022.1038479 (PMC9763934; doi:10.3389/fendo.2022.1038479)
Supplement: Supplementary file 1 [file DataSheet_1.docx]

**Primers’ sequences**

| **Gene name** | **NCBI Accession no.** | **Sequences (5′–3′)** | **Primer pair efficiency** |
| --- | --- | --- | --- |
| ***srebp*** | KJ162572 | F CGTCTGCTTCACTTCACTACTC  R GGACCAGTCTTCATCCACAAA | 98% |
| ***fas*** | KY378913.1 | F CAGCGCCGCCACGACAACCT  R GGCCTCCGCTGGCACAGGAA | 93% |
| ***accα*** | XM_042723589.1 | F CCGGGCCGCATGCAGGAGAA  R TGGCCGTCCGTCAGCTCACTGTT | 101% |
| ***hsl*** | MF061228.2 | F GCCCTGGGCCAGTCAAAGTGC  R CCCTCGGGACGAGAAAGGGCCAA | 94% |
| ***atgl*** | KY906167.1 | F ACAGGATCACTCCAGCATCG  R CCTCCACCAGCGCTTTATGT | 96% |
| ***cpt-1a*** | KP262350.1 | F CGCGAGGGCCGGACGGAAAC  R TCGTGGTGCTCTCGTCCTCCA | 97% |
| ***cpt-1b*** | KJ816746.1 | F GCATAGACTCACCCTTCCTCAAA  R GAATGTCGATGAGATTGAGCTGC | 97% |
| ***igf-1*** | XM_019092966.2 | F TCCCAGGACACCAAAG  R TCGCCCTACTGTCTTCTA | 98% |
| ***pgc-1α*** | XM_042765856.1 | F CTTGCTGCCCTTGAGAATGG  R AGCATGCGCATCTAAGTCTGT | 93% |
| ***glut2*** | XM_042718276.1 | F CCAGCAGCCATCGCATTAGC  R GCACCATCTCAGCCTCTTCTTG | 95% |
| ***hk1*** | XM_042736852.1 | F TGGCGCTACTGCAGGTCAGGT  R AGCCCGGCGGGAAACAGCTC | 94% |
| ***pk1*** | XM_042715494.1 | F AACGACGTGTGGGCCGAGGA  R TGGACGCCAGCCGGTCAGGA | 100% |
| ***pfk*** | XM_042764356.1 | F CACGTACAAGCTGTTAGCT  R TCGAAGCCATCATGGACGGT | 97% |
| ***g6p*** | AF427863.1 | F TGGTTGTTGCCGAGGCCTTCA  R TGGGCTTTCTCCAGGGTCCACAGC | 96% |
| ***fbp*** | AF427864.1 | F TGGTTCTCTCCACAGGCCAAGG  R GGGCGAACTTCCATCCTCTGGGA | 103% |
| ***pepck*** | KP250869.1 | F GGTGCCCTCTTTGACCTGCCCAA  R TCTGGCCTCCAGCGCCCTCA | 97% |
| ***fabp2*** | GU937798.1 | F GTTCCACGTGAAGGAATCCAG  R GTGGTTATTAGCGACTTTCCGTTG | 94% |
| ***cd36*** | KM030422.1 | F CTGATTGCTCGGATGGACCT  R CACCAAGCATCCCTCCCTTTA | 98% |
| ***sglt1*** | JQ767161.1 | F CTAAAGAAGAGGAGGCAGAGTTG  R ACAGACGGTGAGGAGGATAATA | 100% |
| ***pept1*** | JN896885.1 | F TCCAGGCTGGTTGGCTGTTG  R GCACGTATTCAGCCCACTGC | 101% |
| ***lat2*** | XM_042718696.1 | F ACAACCAGATATGCACCGGC  R CCCAGTACACGCCCAAGAAA | 96% |
| ***β-actin*** | JQ619774.1 | F GATGATGAAATTGCCGCACTG  R ACCAACCATGACACCCTGATGT | 100% |

*srebp*, sterol regulatory element-binding proteins; *fas*, fatty acid synthase; *accα*, acetyl-CoA carboxylase α; *hsl*, hormone-sensitive lipase; *atgl*, adipose triglyceride lipase; *cpt-1a*, carnitine palmitoyltransferase 1a; *cpt-1b*, carnitine palmitoyltransferase 1b; *igf-1,* insulin-like growth factor 1*; pgc-1α，*peroxisome proliferator-activated receptor (PPAR) gamma coactivator-1 alpha; *glut2*, facilitated glucose transporter member 2; *hk1*, hexokinase 1; *pk1*, pyruvate kinase 1; *pfk*, phosphofructokinase; *g6p*, glucose-6-phosphatase; *fbp*, fructose bisphosphatase; *pepck*, phosphoenolpyruvate carboxykinase; *fabp2,* fatty acid binding protein 2; *cd36,* Cd36; *sglt1,* sodium/glucose cotransporter 1; *pept1,* oligopeptide transporter 1*; lat2,* y^+^L amino acid transporter 2
