# Supplementary material for: Overexpression of growth hormone improved hepatic glucose catabolism and relieved liver lipid deposition in common carp (Cyprinus carpio L.) fed a high-starch diet
Source: Front Endocrinol (Lausanne). 2022 Dec 6;13:1038479. doi: 10.3389/fendo.2022.1038479 (PMC9763934; doi:10.3389/fendo.2022.1038479)
Supplement: Supplementary file 3 [file DataSheet_3.docx]

**Two-way ANOVA results on interaction between *GH* level and dietary carbohydrate level for measured Parameters of figure 6D (*P* value).**

| Organs | Parameter measured | *GH* | Carbohydrate | Carbohydrate  × *GH* |
| --- | --- | --- | --- | --- |
| Liver | Relative expression of *igf-1* | 0.001 | 0.364 | 0.284 |
| Liver | Relative expression of *pgc-1α* | 0.024 | 0.334 | 0.019 |
| Liver | Relative expression of *glut2* | 0.847 | 0.885 | 0.032 |
| Liver | Relative expression of *hk1* | 0.012 | 0.001 | 0.909 |
| Liver | Relative expression of *pk1* | 0.290 | 0.015 | 0.162 |
| Liver | Relative expression of *pfk* | 0.233 | 0.433 | 0.034 |
| Liver | Relative expression of *g6p* | 0.000 | 0.002 | 0.000 |
| Liver | Relative expression of *fbp* | 0.014 | 0.089 | 0.616 |
| Liver | Relative expression of *pepck* | 0.136 | 0.407 | 0.001 |
