# Supplementary material for: Overexpression of growth hormone improved hepatic glucose catabolism and relieved liver lipid deposition in common carp (Cyprinus carpio L.) fed a high-starch diet
Source: Front Endocrinol (Lausanne). 2022 Dec 6;13:1038479. doi: 10.3389/fendo.2022.1038479 (PMC9763934; doi:10.3389/fendo.2022.1038479)
Supplement: Supplementary file 4 [file DataSheet_4.docx]

**Two-way ANOVA results on interaction between *GH* level and dietary carbohydrate level for measured Parameters of figure 9 (*P* value).**

| Organs | Parameter measured | *GH* | Carbohydrate | Carbohydrate  × *GH* |
| --- | --- | --- | --- | --- |
| Liver | Relative expression of *srebp* | 0.579 | 0.846 | 0.253 |
| Liver | Relative expression of *fas* | 0.683 | 0.128 | 0.093 |
| Liver | Relative expression of *accα* | 0.090 | 0.000 | 0.609 |
| Liver | Relative expression of *cpt-1a* | 0.001 | 0.854 | 0.408 |
| Liver | Relative expression of *cpt-1b* | 0.126 | 0.292 | 0.116 |
| Liver | Relative expression of *hsl* | 0.007 | 0.043 | 0.655 |
| Liver | Relative expression of *atgl* | 0.603 | 0.603 | 0.551 |
