# Supplementary figures and images for: Overexpression of growth hormone improved hepatic glucose catabolism and relieved liver lipid deposition in common carp (Cyprinus carpio L.) fed a high-starch diet
Source: Front Endocrinol (Lausanne). 2022 Dec 6;13:1038479. doi: 10.3389/fendo.2022.1038479 (PMC9763934; doi:10.3389/fendo.2022.1038479)

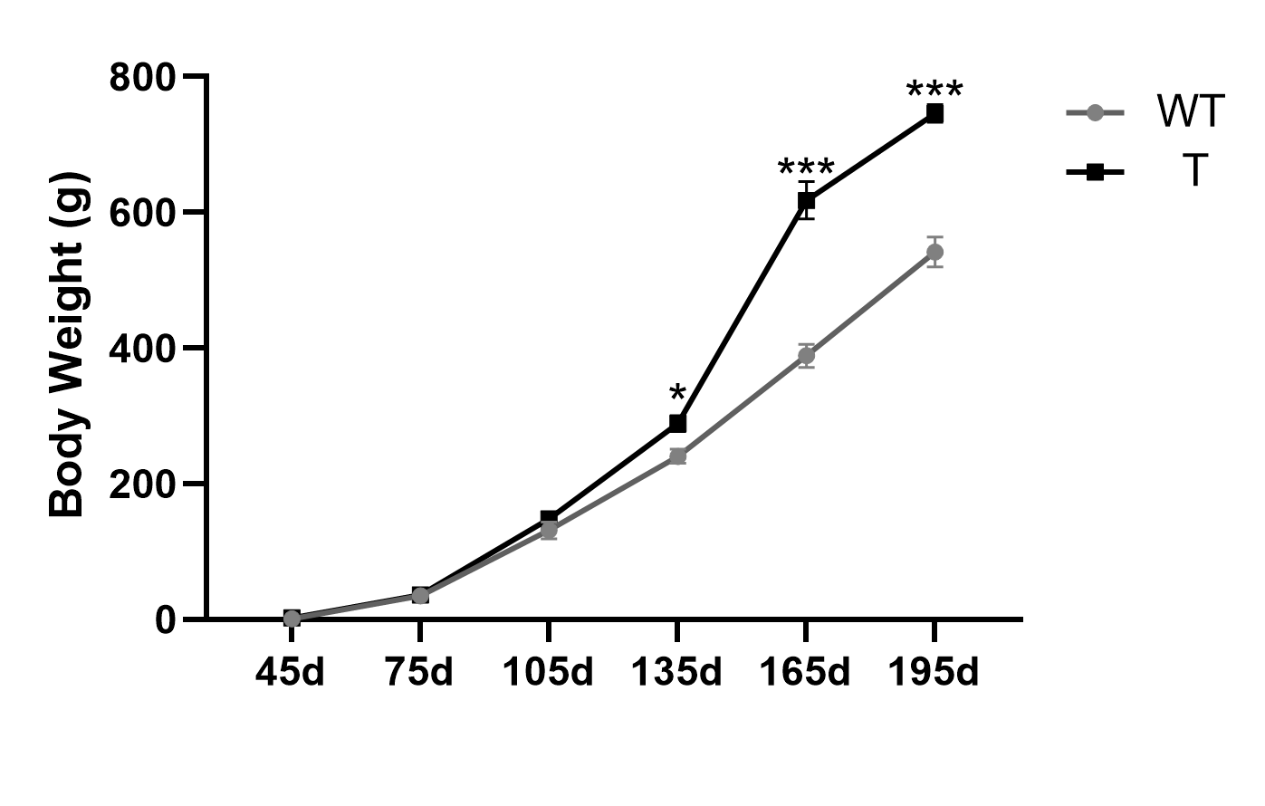


Body weight of WT and T carp from age of 45 days to 195 days (n=8). * *P* < 0.05, *** *P* < 0.001

Supplement: Supplementary file 5 [file DataSheet_5.docx]
